# The Dentist—Guardian of the Gateway to Nutrition

**Published:** 1912-05-15

**Authors:** S. M. Stauffer

**Affiliations:** Pittsburgh, Pa.


### THE DENTIST—GUARDIAN OF THE GATEWAY TO NUTRITION.

BY S. M. STAUFFER, D. D. S., PITTSBURGH, PA.

The proper use of food is logical, but to indulge in foods when sick, under the vain thought that it will "keep up strength," even though there be no hunger, is a violent violation of Nature's laws. Such signals as lack of desire should be observed carefully, for they are warnings that she has gotten behind in her work and demands a chance to catch up. Food taken under these circumstances is but partly digested —if at all— and ferments and decomposes, producing auto-intoxication. Dr. Dewey said: "Take food away from a sick man's stomach and you have begun, not to starve the sick man, but the disease."

If we are to be Guardians of the Gateway to Nutrition, we must have knowledge of the value of foods as *food*, and to know how to aid and direct patients in making proper selections, together with their preparation. This requires an extension of knowledge beyond the mere filling of teeth or the making of artificial substitutes. But, once in possession of the knowledge, it simplifies living to an extent by reducing the drudgery of the culinary department by more than half and to such as are interested, from a purely mercenary point of view, it *must* commend itself—especially so since the enjoyment of the food will be an unusual delight to the most fastidious epicure. If any one of you is inclined to doubt this statement, let him but wait for true, physiologic hunger to assert itself; then note with what gustatory delight the simplest foods will be relished.

If we are all leading a normal life, spending plenty of time in the open air, Fletcherizing our foods, not using all of our stored-up energy in "the strenuous life," having peace of mind, perfect pois, harmonious surroundings, plenty of fresh air and diaphramatic breathing, exercising in plentiful and pleasurable form, eating only when physiological hunger asserted itself—to satisfy hunger, not appetite—then we would likely select our food with intelligence, and the matter of nutrition should need but little attention.

Unfortunately, however, this is the age of conventionality. The conventional life of the past, with its complicated feast-day menus and the illogical and too frequent hours of food indulgences, has ruined the teeth of mankind and rendered the existence of our profession necessary. Little did Benjamin Franklin realize the import of his saying, 170 years ago, that "America was noted for her poor teeth, and as a soup-eating nation." This remark certainly implies that they were, even then, eating of foods much too soft; not well selected, nor prepared in the manner required for use in the development of dental organs. And conditions have grown steadily worse, until within a decade or two there have developed changes in some of our methods of thinking and doing.

Cooks, in their imagined wisdom, have for years been soaking and parboiling foods and throwing away the *mineral* food salts, under the impression, no doubt, that they were getting rid of the "non-nutritious"—the injurious elements. Whereas, the truth is, they have been soaking and parboiling out and throwing away the salts most needed in foods, and all this done, no doubt, with the notion that it added to the "gustatory delight." In cooking foods, none of the water should be thrown away. Many of the foods so treated are the ones richest in soda and lime, the mineral food-salts in which so many foods are deficient. Those rich in soda and lime salts which are treated thus are cabbage, spinach, onions, beets, dandelion, stinging-nettle, cucumbers, potatoes, asparagus, rice, etc. Meat, too, is soaked by some people until much of its food-salts are lost. This should not be done *if you are going to eat the meat!*

One of our recent philosophers, Horace Fletcher, has done more, in my opinion, to help mankind in its present deplorable, debauched condition than any other living mortal. In our present manner of eating we gluttonize. We do not eat-to-live, as we should, but we make ourselves a close second to that omnivorous creature which eats, grunts and squeals and then hangs, head down, in the market stalls. And in consequence a large portion of the food we eat only ferments and decomposes—rots—in the alimentary canal, the results of which are much distress and suffering. We call this *disease.* Yes, dis-ease, not at ease.

When I say "debauched" I don't mean "wallowing in the gutter," as is so frequently meant in reference to alcoholic inebriates. But I do mean we create troubles brought on by intemperate habits of eating which, in reality, are just as degrading as those of the individual who indulges in the gutter escapade. In making this statement I do not except those who pose as the elite of society, nor the learned doctor; for they, as we, succumb to violations of natural law—just as surely as a law is broken. I might go on at this rate indefinitely. But time forbids.

Regarding your duties as Guardians of the Gateway to Nutrition, you may ask: "Where do they begin?" They begin with the little child in your chair; for it is hard to teach the "old dogs new tricks." Teach the children the importance of the dental organs and how to care for them—physiologically as well as prophylactically—which latter word is here used because *you* use it and because it "sounds" better—more "professional"—than the real word "sanitary," which ought to be used. I dare say that had you, when a child, been taught to care for the teeth, physiologically (and this includes both prophylaxis and sanitary means), to select foods offering resistance enough to be cleansing and to exercise the teeth and gums sufficiently to develop them into strong, healthy organs, together with the use of brush, floss and picks, you would be happier, and have better teeth.

Go back one week in your memory and note if you have, had at any meal, enough solid, resisting food which would in its excursions over the sides of the teeth and gums drive the colonies of those apparently harmless emigrants from their camp. The manner of preparing foods today would, in reality, enable us to get along without teeth—were it not for the necessary mixing-in of the saliva. Hence, the time seems ripe for an awakening to the facts. Ascertain for ourselves, then instruct our patrons in the importance of caring for their dental organs—not alone with reference to sanitation, but in the manner of using them for the purpose of getting *nourishment*—not auto-intoxication—from the foods they employ.

To become a Guardian of the Gateway to Nutrition, it is necessary to know the *reasons* for such guardianship—the *reasons* for decaying teeth—other than the supposed "purely bacterial" ones; the *reasons* for the secretions of the mouth becoming so abnormal that caries are inevitable—despite the lavish indulgence in "antiseptic" washes and powders; the extremest care, personally, as well as at the hands of a competent dentist.

To find these *reasons* and remedy them will be doing a vaster good for mankind than the discovery of an invisible, indestructible and painlessly inserted "filling material."

These *reasons* may be discovered in a comparative study of life in the evolution of our race. The history of our barbaric ancestors dates back many years. What was their diet? We know this sufficiently well, through study of anthropology. From the shape of our teeth, many have formed the opinion that we are frugivorous; some carnivorous, and others omnivorous. The teeth, however, play, the least part in determining this point. Study will reveal that the mammary glands, the colon, the tongue, the skin, the nails, the salivary glands, the teeth, and the attitude in walking, all play an important part in deciding this important question.

There is no doubt, however, that our teeth do belong to the type of the frugivorous; animal; and, from the proofs available, it is certain that the diet of our remote ancestors was chiefly fruits and nuts.

We have no knowledge as to the extent to which meats were used as foods, but in times of scarcity they may have resorted—as the horses of the northwest Norwegian coast have been known to do—to plunge into the sea to catch fishes when driven to this extreme by starvation.

As to the characteristics of the mineral salts in a diet of fruits and nuts we find, on examinations of the ash, from five to ten times the quantity of soda and lime, as compared with that found in the diet as prepared for man today. The other mineral salts are about in the normal proportion.

In view of the fact that our diet is so woefully lacking in the soda and lime salts, is there any real wonder in the degeneration—caries—of tooth structure?

In some cases of osteomalacia (a morbid softening of the bones) the deficiency in soda and lime is found to be 60% below normal.

The cause of rickets and rickety teeth is now known to be largely due to diet, deficient in mineral salts.

It can hardly be doubted, either, that the early loss of the third molars, the ugly, soft, yellow crowns of the other molars and bicuspids and the general lack of "resistance" in so many teeth cannot be due to causes other than an insufficient supply of the lime salts.

These statements are based upon facts gleaned from "Natural Hygiene," by H. Lahmann, M. D., and I believe them to be the keystone of the situation.

Note the fact that the babe at the breast gets milk only fair in soda and lime. Whereas, the Indian babe—the papoose—is fed at the breast until it is nearly three years old, and decayed teeth amongst these people is very rare.

In artificial feeding the milk or cream is diluted from two to four times, thus lessening the quality of food salts to about 25% to 50%.

Now you will ask me to define the diet which may give us the normal amount of food salts. What I have to say shall be general in its application, because—even to those who may have no teeth—it is a factor in the prolongation of youth—the maintenance of health.

In the first place it will be necessary to set aside our "conventional menu" and adopt one of those recommended by Drs. Tilden, Lahmann, Bunge, or any of that class of naturalists, who condemn the too prevalent use of meats broths, eggs, starches or cereals stimulants, pulses, cheese, etc., and in their stead eat, almost exclusively, of green leaf and root vegetables, green salads and juicy-fruits.

You will recognize the fact that the raw—or sun cooked—vegetables and fruits are of the utmost importance in the development of the teeth and jaws of children, which is very significant, especially if the food be well selected with regard to the "food" or mineral salts. These foods are firm in texture and require more careful attention in the matters of mastication and insalivation than can be the case of the cooked, starchy and soggy foods our tables chiefly abound in.

If we look up the diet of our prehistoric ancestors, we will discover that it consisted of meats, nuts, tropical fruits, succulent roots, leaves and vegetables, wild honey and, may be a few other simple, uncooked foods. There was no bolted flour; no cooking and parboiling, then carefully draining off the best portions and eating the *residue*, which is done in our enlightened civilized homes today. This, with their "outdoor life," constituted their "simple mode of life."

If we compare their diet with our own, observing the difference in the quantity of food salts, we find a definite contrast, accounting possibly in a large degree for the definite shortening of life's span, as shown by the insurance companies tables. Another disadvantage, too, under which we labor is in the fact that our foods are all prepared for us by "skilled" cooks, who possess no knowledge of the value of combinations other than for the mere production of flavors to pander to "appetite."

I want to call your attention to a few points which will be well for us to consider if we are to live in accordance with "the best knowledge obtainable today." It is true we are sadly in need of knowledge whereby we can better control our physical conditions; but if we will use that which is available we shall do much better than can be possible in the "conventional" life which most of us have been "brought up to."

Many of you no doubt think our present diet is a recent invention; be that as it may, we may judge from what Benjamin Franklin said, that there was something radically wrong in its selection and combination nearly two centuries ago. Why? Simply because they were living the "conventional life"—out of balance in a variety of ways.

In such a diet as was used by our prehistoric ancestors the food-salts were well balanced. Compared with the diet of today, there is a vast difference. Our diet contains excesses of potash, iron, sulphuric acid, etc., and is deficient in soda, lime, phosphoric acid and chlorin.

Gardeners, farmers, and stock raisers are thoroughly drilled in all their specialties except in the raising of their children properly. Hygiene should be taught in a manner easily understood by tots even. You might think it hard or even impossible, but I want to say the child is easily taught and will grasp the subject securely if given the opportunity. The trouble, however, is that "grown up" children—those with the gray hairs— are deficient in their knowledge of these things, and as a natural result there is improper feeding of the younger generations; hence, epidemics rage and mankind suffers. Plant life also suffers from want of proper mineral substances.

Dr. J. H. Tilden of Denver, Col. (whom Dr. Charles A. Du Bois of New York considers the greatest living authority on dietetics) says if the Southern people afflicted with "hook-worm" will cease the use of pork and use only a reasonable amount of cream or refined cottonseed oil for shortening their bread, then see to it that nuts, eggs, chicken or fish are used to the *exclusion* of other meat foods and *never eaten oftener* than once during a day, in conjuction with a dinner plateful of salad made by combining lettuce, cabbaage, celery and tomatoes—dressed with a little salt, sugar and *lemon juice*—not vinegar; and one meal a day of fruit, cheese and milk, "I will guarantee there will be no hook worms in the intestines of those who adopt such diet within one year after its adoption." But *their fields must be fertilized* with something containing potash, soda, lime and magnesia, as their soil is in a state of degeneration.

Neither soil nor the people can be cured by dosing with thymol and salts. Give back to the earth the elements necessary for "man-building" and she will go out of the hook-worm business.

In healthy organisms fat is formed from the fats in food and from carbohydrates; while, in morbid conditions, the *incomplete* oxidation of albumen seems to be the principal source of fats, as is shown in the accumulations of abnormal urin constituents, and explains why fat persons are more subject to kidney diseases and gout.

Lean persons worry themselves in their endeavors to get stouter, by eating and drinking, but fail. Why? The following experiments, by Foster, will illustrate: He fed three pigeons white bread and casein, either of which contains few food salts; they lived thirteen, twenty-five and twenty nine days, respectively. He next fed two dogs on meat deprived of its food salts (by soaking), some fat, sugar and white bread; the dogs were at the point of death on the twenty-sixth and thirty-sixth days. Dogs, completely deprived of food, will live from forty to sixty days.

Dr. J. H. Kellogg, Dr. T. J. Allen and Mr. Bernarr Macfadden have each experimented with dogs, apparently under the same conditions, feeding one with bread made from *bleached* white flour; one with bread made from white flour, and one with bread made from the "whole-wheat" flour while a fourth dog was fed nothing at all. The dog fed with bleached flour bread died first; the one fed with white flour bread died next; the one fed nothing died third, while the dog fed with whole wheat flour bread apparently thrived—did well.

The process of bolting flour removes the mineral, or food salts. Bunge proves this by means of experiment. He shows, also, that the sulphuric acid, formed from the sulphur of albumen, finding no food salt basis to combine with, destroys the living cells.

The treatment of bad cases of lean dyspeptics is difficult; it requires a long time. And to obtain results they must give up their senseless notions concerning "generous diet," meat, broths, eggs, beer, pulses, cereals, cheese, etc. Such cases will not improve until they can obtain proper quantities of soda and lime with their food, which should consist largely of green vegetables, green salads and juicy fruits.

It is difficult to make people understand that the same diet can produce corpulency in one person and "leanness" in another, but experience teaches that this is actually the case—one person possessing a more active heart than the other. In one case the blood is diluted, owing to the weaker action of the heart, and the albumen is transformed into fat, and in this form protects the tissues from attacks of acids. With the stronger heart's action the reverse is the case.

If uric acid is a prominent factor in the development of disease, may it not be probable that it interferes with the development and aids in the destruction of the teeth? I most certainly think so.

It is a fact well worth considering that the urine of carniverous animals—such as the dog and cat—is, usually, quite free from uric acid; whilst that of the human animal varies, according to the food taken. If vegetable food alone be consumed the urine will, like that of the herbivorous animals, contain only traces of uric acid.

Man is the only animal existent which suffers from uric acid troubles. Could this arise from any cause other than the wrong choice and use of foods?

According to Landois and Sterling, the amount of uric acid daily excreted through the kidneys is 32.5 grains on a flesh diet, and from 3 to 10 grains on a non-flesh diet.

When we recognize the fact that uric acid is a product of imperfect nutrition, that it is the result of stuffing the body with excessive nitrogenous wastes, the figures above become more than significant. If a meat diet increases the amount of uric acid found in the urine from three to ten times that resulting from a simple, natural diet, is not the question well worthy of careful, earnest consideration?

Our present "practice of medicine" consists, almost wholly, of "*treating the symptoms*"—rheumatism, skin diseases, nervousness, etc., etc. The lack of any system which treats the *cause* of disease; the want of a proper *system* of prophylaxis; the want of *success* in the present system of therapeutics, and the rapid growth of "quack medicine" are all explained by our simple ignorance of the *cause of disease;* call it "primary" or what you choose—it's still the *cause*!

Animals have preserved their instincts with reference to choice of foods. Man has the disadvantage of "cultivated" (?)tastes; not alone with reference to tobaccos, liquors, opiates and condiments, but *foods*—their ill-judged mixtures as well as their ill-timed indulgences.

We know that the food of man should contain certain amounts of albumen, fats, carbohydrates and inorganic salts. Of the three former elements the amounts have been pretty well determined. But the man who eats albumen, fats or carbohydrates alone or in proportion inadequate to the needs of the body will sicken and die. So, also, would he sicken and die if he ate chiefly or if he avoids the soda, the lime or the iron salts.

A great many imagine, when speaking of food salts, that the common chloride of sodium (table salt) is referred to. This is not at all the case; nor does this grade of "salt" aid digestion, form bone or perform any other physiologic gymnastics. It is one of the natural *excretions* of the human body and need not be taken, in its "manufactured" form, for the only use to which it can be put—excretion!

Our unphysiologic living is well shown in what Dr. Ellen Goodell Smith said: "There is not a farmer in all the land who would dare to feed his cattle on the impoverished food he places upon his own table."

I wish to say that we have departed so far from "Nature's Laws," which are "God's Laws," by our criminally erratic conventional habits and customs that we have lost our instinct in the matter of choice and preparation of food. If our intuitive faculties—good sense—were in reasonably active order, there should be no such thing as disease. Health and supreme happiness should reign. Our instincts, however, were lost long before we were able to creep. Even at the moment of our birth the nurse or the mother, who have been tutored in the violation of Nature's laws, begin the spreading of "bad habit" by premature feeding; and insist upon its early and complete cultivation by incessant repetitions at intervals of *two hours*—thinking she knows "Nature's Laws" better than the innocent naturalist (which it would be if left alone). What is the natural result? Food instinct is frightfully interfered with, and finally lost. Dr. C. E. Page of Boston said: "If the meddlesome mothers would only leave the babies alone, with regard to their feedings we would have to do with them as we do with surplus kittens and puppies—drown 'em!"

Dr. Page advocates the feeding of babies not more than three times daily. At birth is the period of life when the child should be "started right." It is then the foundation of temperance or intemperance in all things is laid; and intemperance in the matter of food started at this period of life and continued (which is I think the greatest error committed by mankind) warps the bodily and mental structures, so that disease or crime are the natural developments. And crime is only an expression of disease—*not anything else*!

Dr. Page lost his first wife and three children. Realizing something was wrong in the method of treatment, or elsewhere he began the study of medicine; then Nature's "cures." Afterwards he re-married and has had five children, ranging in age from five to sixteen years, during which periods not one of them has experienced a day's sickness—not one of them was drilled to such torture by a cultivated habit of over-eating!

If all children were raised like those of Dr. Page the dental and medical professions, as now existent, would waft kisses of farewell from their hands ere they shifted to more needed fields of usefulness!

Oral hygiene, as now heralded, is a noble movement on the part of an unselfish profession. It realizes the distress of suffering children, and I glory in the desire to aid and relieve this dire and depraved condition of the little ones. It will help them physically, mentally and spiritually—spiritually away beyond the fondest expectations of the instigators of the movement. But you are looking only at one phase of this vast subject. It is not charitable to supply the needy with money and food. Rather is it more of a charity to place them in a position where they may earn such money and food as they require, without expecting it to be handed them without personal effort upon their own parts. Hence, I say, continue the "Oral Hygiene" movement if you like it; *but*, for for the sake of suffering humanity, let us strive to get at the root of this great evil and see how nearly we may come to correcting it. "Impossible?" Yes, it looks that way, especially since our own beloved profession, our medical colleagues and our learned professors in the arts and sciences know little or nothing of the matter of building good constitutions—good teeth. They have never given this feature of the subject a thought—beyond their own desires to gluttonize and gourmandize, with blind faith hitched to drug stores.

Now, I have a proposition to make: Inform yourselves upon this subject in some manner. If you cannot find it in books, create it! Dig it up somewhere—by personal experiments. A little conscious awakening, a little determination—a start, and the world may be revolutionized by you!

I have suggested to you, from our dental standpoint, the deficiency in soda and lime. Suppose you do, as did Dr. Lahmann, "try it on the dog." Of course, the "human dog" is rather inclined to fear "experimenting" upon himself, but how else may we determine the value of foods? Of course, if there be upon the table (and we indulge in them )from half a dozen to twenty—and sometimes more—different articles of food, we may expect no results other than those with which we are and have been accustomed to.

While in New York, on one occasion, I visited B. Lusts' bakery, where they said they burned their stale whole-wheat bread. In reply to my query, "Why don't you give it to the army of poor people surrounding you?" they replied: They won't eat it! This is what they want," pointing to a mass of cheap, sugar-coated cakes and pies.

The place to start this reform is in the public schools. Teach the pupils the most desirable foods for the upbuilding of good teeth; tell them how to live, to avoid anger, worry and depression, which interferes seriously with the physiologic functions—digestion, assimilation, and elimination—the healthful maintenance of the whole body, and in time, however long it may prove to be, we shall have a race of people with *good teeth*.

I can think of nothing of any more importance than to teach these children how to select and prepare their food as referred to above.

A matter so eminently practical must also be treated practically.

So important a matter as feeding, the foundation and mainspring of bodily and mental, individual and social health, deserves the best work of the highest minds.

### DISCUSSION.

Dr. Tufts: The facts presented by Dr. Stauffer seem to me to be fundamental in the cause of dental caries. By comparison, oral hygiene and prophylaxis is but a crutch for the present generation, and will have but a minor effect in the evolution of the teeth of mankind, while we know what a major factor diet is in the development of animals and man, especially such a change as a great reduction in the salts of soda and lime.

We know that the diet of the race for 300,000 years was game, fruit, nuts, with succulent roots and leaves. The ash of such foods in any likely combination contains an abundance of all mineral substances required by a normal body. The average diet of today is dificient in the salts of soda and lime, containing but from 20 to 50 per cent. of that of our barbarous ancestors.

The skulls of even the 17th century show full sets of teeth. The greatest change which has occurred in diet since this time is a vastly increased consumption of decorticated cereals, which were already deficient in lime and soda, which is so weakening to the system that they now recognize the use of polished rice as the main factor in the cause of beri beri disease. Evidence of the bad effects of a deficiency of mineral salts is rapidly multiplying.

These facts are to me very striking, and I think we are now beginning to reach some of the broad foundation stones of the cause of dental decay.

To get the necessary food salts we should eat the whole grain and more fruit and green vegetables, reducing the amount of cereal products. At least one meal a day should be vegetables and fruit, without any starchy food.

The best foods to balance a diet in this respect are spinach, lettuce, asparagus, cabbage, apples, grapes, oranges, peaches and pineapple.

Dr. Stauffer: I am not a vegetarian, although I believe we would all be better off without meat.

Our intemperance in eating is not the result of human nature, but of our bad habits. Give Nature a chance by abstemious living or fasting to burn up the clinkers and make new blood, and she will do it. If the thin person who eats too much will eat (for a period) little enough he will begin to gain weight, that is if he has a reasonable selection of food, rich in mineral or food salts.

In answer to Dr. Rinehart's question, would say that if you had not fed your typhoid fever, you would have been out of bed in very much less time. I have known cases of typhoid fever to burn out in much less time. I don't believe in *feeding in any fever*, for a fever is produced by Nature getting rid of an excess of fuel.

Children need foods that will build tooth tissue, and should not be deprived of the mineral salts in food. Dr. Black tells us that tooth structure does not change, and we will cheerfully accept Dr. Black's opinion because we have no better authority.

To summarize, dental caries is caused by abnormal living, which causes an abnormal functioning of the body, which produces abnormal oral secretions.—*The Summary*.

